# The many substrates and functions of NEDD4-1

**DOI:** 10.1038/s41419-019-2142-8

**Published:** 2019-12-02

**Authors:** Xi Huang, Jing Chen, Wen Cao, Li Yang, Qingxiao Chen, Jingsong He, Qing Yi, He Huang, Enfan Zhang, Zhen Cai

**Affiliations:** 1 Bone Marrow Transplantation Center, Department of Hematology, The First Affiliated Hospital, School of Medicine, Zhejiang University, Hangzhou, China; 2 Center for Hematologic Malignancy Research Institute, Houston Methodist, Houston, TX USA; 3 Institute of Hematology, Zhejiang University, Hangzhou, China

**Keywords:** Tumour biomarkers, Ubiquitylation

## Abstract

Tumorigenesis, tumor growth, and prognosis are highly related to gene alterations and post-translational modifications (PTMs). Ubiquitination is a critical PTM that governs practically all aspects of cellular function. An increasing number of studies show that E3 ubiquitin ligases (E3s) are important enzymes in the process of ubiquitination that primarily determine substrate specificity and thus need to be tightly controlled. Among E3s, neural precursor cell expressed developmentally downregulated 4-1 (NEDD4-1) has been shown to play a critical role in modulating the proliferation, migration, and invasion of cancer cells and the sensitivity of cancer cells to anticancer therapies via regulating multiple substrates. This review discusses some significant discoveries on NEDD4-1 substrates and the signaling pathways in which NEDD4-1 participates. In addition, we introduce the latest potential therapeutic strategies that inhibit or activate NEDD4-1 activity using small molecules. NEDD4-1 likely acts as a novel drug target or diagnostic marker in the battle against cancer.

## Facts

- NEDD4-1 exerts dual roles as an oncogene as well as a tumor suppressor in cancers.
- The enzyme-dead NEDD4-1-C867S mutant suppresses the function of the NEDD4-1 E3 ligase.
- NEDD4-1, which contains C2, WW, and HECT domains, interacts with distinct substrates separately.
- NEDD4-1 preferentially ubiquitinates substrates through K63 conjugation compared with K48 conjugation.

## Open questions

- Which domain is responsible for each substrate interaction?
- What is the specific function of NEDD4-1 in different tumors?
- What is the new ubiquitination site in NEDD4-1?
- What are the new substrates of NEDD4-1?
- How can NEDD4-1 be targeted to conquer disease?

## Background

Ubiquitination modulates a large repertoire of cellular functions and requires an E3 ligase, which determines the specificity of ubiquitination by mediating the transfer of ubiquitin (Ub) to substrates. In 2004, Ciechanover, Hershko and Rose won the Nobel Prize in Chemistry for the discovery of Ub-mediated protein degradation. In recent years, an endless series of studies on E3 Ub ligases (E3s) have emerged. A large number of studies have confirmed that E3s are closely related to tumorigenesis^1–3^, development^4,5^, metastasis^6^, and prognosis^7–9^.

In this review, we briefly present the process of ubiquitination and the structure of neural precursor cell expressed developmentally downregulated 4-1 (NEDD4-1). Then, we describe some of the substrates of NEDD4-1-mediated ubiquitination and the effect of their interactions with NEDD4-1 on tumorigenesis and beyond. Finally, we simply summarize the clinical relevance of NEDD4-1.

## The manner of ubiquitination

Ubiquitination, a post-translational modification (PTM), usually follows a highly ordered series of enzymatic reactions involving E1, E2, and E3s and targets proteins for degradation or brings about other cellular fates, such as the regulation of enzymatic activity, inflammatory signaling, endocytosis, and histone modifications and has been shown to be involved in numerous cancers^10–13^. Ub contains seven lysine residues (K6, K11, K27, K29, K33, K48, and K63), one C-terminal glycine (Gly) site and an N-terminal methionine residue (Met1). The K48 linkage is involved in proteasome pathway-related ubiquitination, while the K63 linkage is involved in DNA repair, protein trafficking, autophagy, immunity, and inflammation, among other processes. These lysine residues are both poly-ubiquitinated, forming multiubiquitin chains^14^. In contrast, the processes by which a single Ub is attached to one or several lysine residues is called mono-ubiquitination. This modification regulates membrane transport and transcriptional regulation^15^. Upon different types of ubiquitination, substrate proteins selectively marked by Ub are degraded by the Ub-proteasome system (UPS), activated or transported^9,16,17^.

## Structure of NEDD4-1

**Fig. 1:**
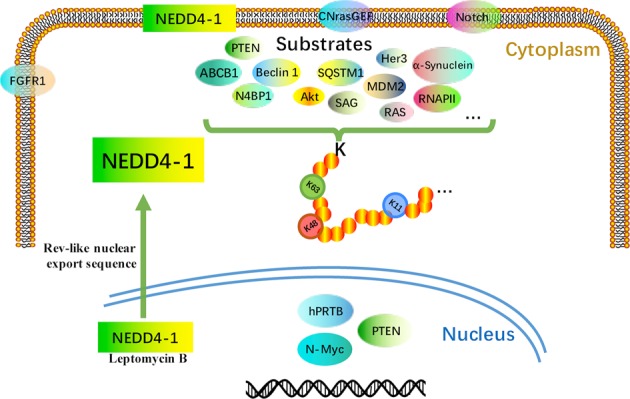
Localization of NEDD4-1 and its substrates. NEDD4-1 is mainly localized in the cytoplasm and can also be localized in the cell membrane, nucleus, and exosomes.

**Fig. 2:**
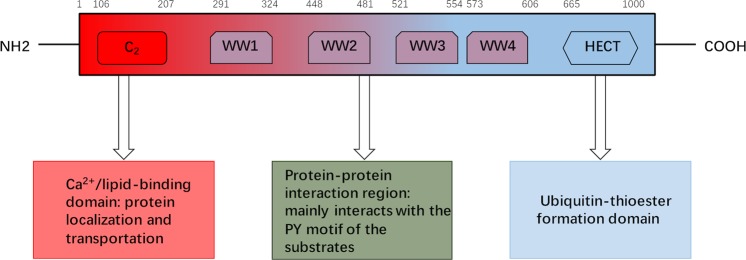
Schematic diagram of the structural features of the NEDD4-1 protein. The NEDD4-1 protein mainly contains three domains: (1) the C2 domain (2) the WW domain, and (3) the HECT domain.

NEDD4-1, also known as NEDD4 and RPF1, was first isolated in 1992 from mouse neural precursor cells whose mRNA levels were downregulated during mouse brain development^18^. Many eukaryotes have several NEDD4-1 or NEDD4-like E3s that seem to have both dismissed and particular functions, but *S. cerevisiae* expresses only a single NEDD4 family member, Rsp5p^19^. The human NEDD4-1 gene is located on chromosome 15q21.3 and contains 33 exons that encode a protein with a molecular weight of ~120 kDa. The NEDD4-1 protein is predominantly localized in the cytosol, mainly around the nucleus. NEDD4-1 is also recruited in the exosomes by the NEDD4 family interacting protein Ndfip1 (Fig. 1)^20,21^. The NEDD4 family contains nine members in humans: NEDD4-1 (also known as NEDD4), NEDD4-2 (NEDD4L), ITCH, WW domain-containing E3 ubiquitin protein ligase 1 (WWP1), WWP2, NEDL1 (HECW1), NEDL2 (HECW2), SMAD-specific E3 ubiquitin protein ligases (Smurf1) and Smurf2^22^. These nine NEDD4 family members are highly conserved E3s in evolution, and each contains a C2 (Ca^2+^/lipid-binding) domain, 2-4 WW domains, and a HECT domain (Fig. 2)^23^. The C2 domain mediates the binding of NEDD4 to the membrane and participates in the recognition of substrates. The WW domain is named for its two tryptophan (W) residues and serves as a protein-protein interaction region that interacts with the PY(PPYY) motif or phospho-serine/threonine residues of the substrate protein. Therefore, although the structures of NEDD4 E3 ligases are similar, they have different functions due to their different WW domains^24^. The HECT domain is a conserved C-terminal catalytic domain that possesses the intrinsic enzymatic activity of NEDD4-1. Structural studies have shown that the HECT domain is composed of two architectural features (the N-terminal (N) lobe and C-terminal (C) lobe); the N lobe of HECT interacts with E2s, such as UbcH5b^25^, while the C lobe contains a catalytic cysteine for transient Ub-thioester formation^26,27^. In addition to intermolecular interactions, the three C2, WW, and HECT domains interact and affect the activity of NEDD4 E3 ligases. Intramolecular interactions, such as those between the HECT and WW domains (NEDD4-1, NEDD4-2, ITCH, WWP2) and the HECT and C2 domains (Smurfs), inactivate NEDD4 E3 ligases^9,28^. WW domains and peptide linkers binding WW domains can lock the HECT domain, leading to the inactivation of the HECT enzyme and autoubiquitination of E3s if the brake is completely removed^29–31^.

NEDD4-1 may participate in many human cellular functions because of its ubiquitous expression in the placenta, liver, thyroid, skin, endometrium, gall bladder, urinary bladder, and kidney^32^. More importantly, NEDD4-1 has been reported to ubiquitinate substrates through both K48 and K63 conjugation, but as NEDD4-1 is a HECT domain-containing E3, K63-type poly-ubiquitination by NEDD4-1 frequently competes with K48-type poly-ubiquitination on cellular substrates with linkage preferences^33–35^. Notably, NEDD4-1 can also mono-ubiquitinate its substrates and catalyze the lesser-known K6- and K27-linked poly-ubiquitination^36–39^, suggesting that NEDD4-1 plays multiple regulatory roles via mono/poly-ubiquitination and diverse linkages.

**Fig. 3:**
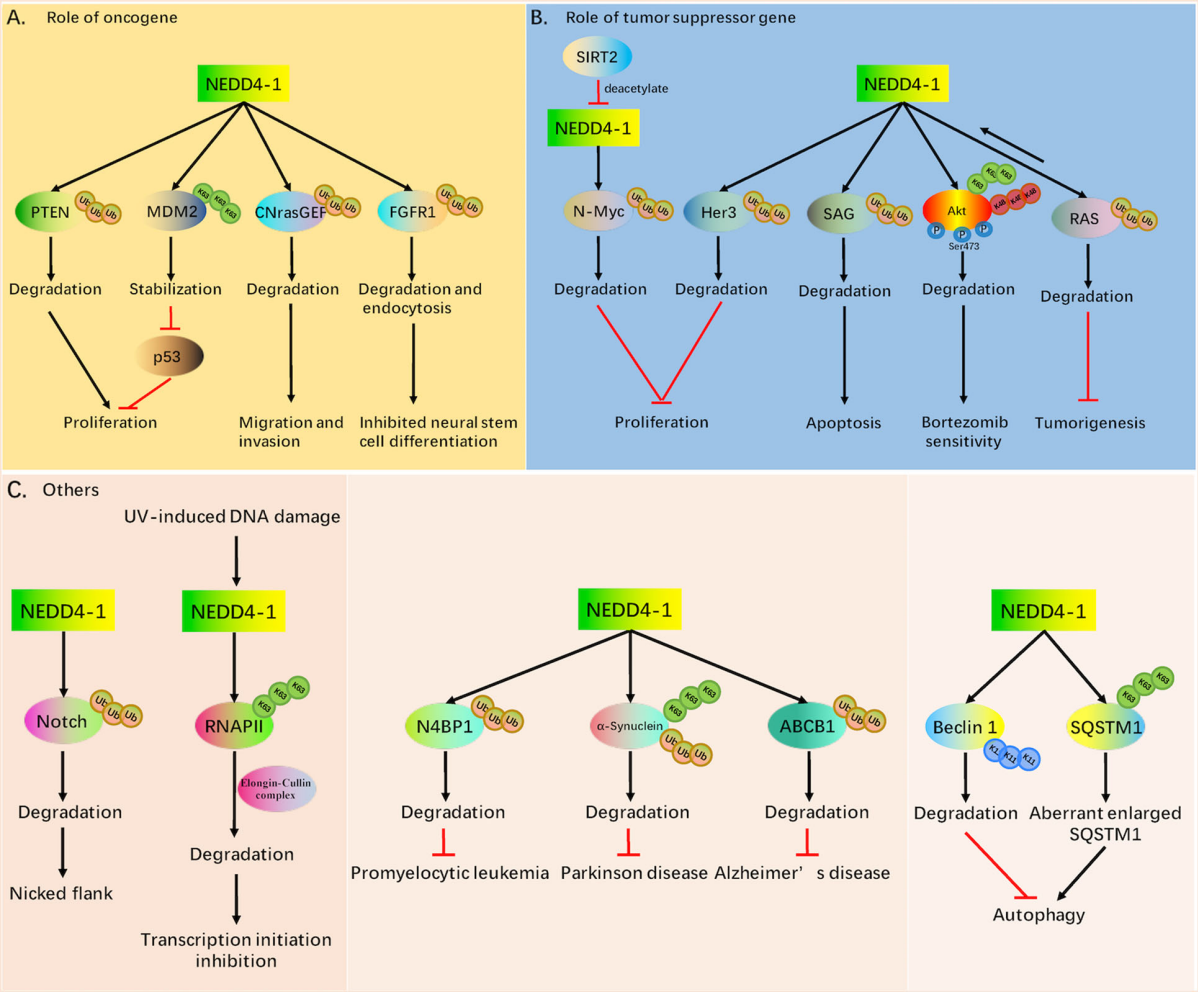
NEDD4-1 mediation of biological processes and other processes.

NEDD4-1 was first thought to regulate the water-electrolyte balance by controlling the abundance of sodium channels in epithelial cells. Subsequent studies gradually revealed that NEDD4-1 acts as an E3 ligase that regulates embryonic development and animal growth (proliferation, autophagy, and the differentiation of multiple malignancies) (Fig. 3). NEDD4-1 has a large number of upstream and downstream genes, and its dual role in cancer makes it a possible molecular switch to regulate tumor development through these competitive substrates. In this regard, some websites (such as STRING and UbiBrowser) and previous discoveries will accelerate research efforts on substrates^40,41^. In the next section, we introduce the substrates of NEDD4-1 (Table 1) and the possible application of NEDD4-1 in cancer therapeutics in detail.

**Table 1:** Substrates of NEDD4-1-mediated ubiquitination.

Targets	Ubiquitination type and linkage	Ubiquitination effects	Functional alteration by ubiquitination or significance	References
PTEN	mono-ubiquitination	PTEN nuclear importation and protected from cytoplasmic degradation	Apoptosis and tumor suppression	^49^
	Poly-ubiquitination	Degradation (proteasome pathway)	Proliferation and tumor Formation; Promotes terminal branching of retinal axons	^50,52,56^
	Independent of Nedd4-1 presence	/	/	^53^
MDM2	Poly-ubiquitination (K63-type)	Stabilization	Proliferation and less DNA damage	^33^
CNrasGEF	Poly-ubiquitination	Degradation (proteasome pathway)	Promoted migration and invasion of glioma cells	^66,68^
FGFR1	Ubiquitination	Degradation and endocytosis	Inhibited neural stem cell differentiation	^72^
N-Myc	Poly-ubiquitination	Degradation (proteasome pathway)	Suppressed neuroblastoma and pancreatic cancer cell proliferation	^78^
Her3	Poly-ubiquitination	Degradation (proteasome pathway)	Inhibited cancer cell proliferation and tumor growth	^82^
SAG	Poly-ubiquitylation	Degradation (proteasome pathway)	Sensitized cancer cells to etoposide-induced apoptosis	^28^
Akt	Poly-ubiquitination (K63-type and K48-type) and multi-mono-ubiquitination	Transportation pAKT to perinuclear regions and degradation of pAkt-Ser 473 but not total Akt	Regulated nuclear trafficking of the activated form of AKT and enhanced bortezomib sensitivity	^87^
RAS	Poly-ubiquitination	Degradation (lysosome pathway)	Suppressed cell transformation and tumorigenesis	^93^
Notch	Poly-ubiquitination	Degradation	The first finding of a NEDD4-1 substrate in muscle; a nicked flank phenotype	^95–97^
RNAPII	Mono-ubiquitination and Poly-ubiquitination	Degradation (proteasome pathway)	Transcription initiation inhibition	^102,103^
N4BP	Mono-ubiquitination and Poly-ubiquitination	Degradation (N4BP1-2) (proteasome pathway)	Inhibition of promyelocytic leukemia	^37,104^
α-Synuclein	Mono-ubiquitination and Poly-ubiquitination (K63-type)	Degradation (lysosome pathway)	Protected against the pathogenesis of Parkinson disease	^107^
ABCB1	Poly-ubiquitination	Degradation	Inhibition of Alzheimer’s disease	^109^
Autophagy-related proteins: Beclin 1 LC3 SQSTM1 (p62)	Beclin 1: poly-ubiquitination (K63 and K11-type)	Degradation (proteasome pathway)	Inhibited autophagy and cell survival	^114^
	LC3: LC3 binds to NEDD4-1, but is not a ubiquitination substrate of NEDD4-1	/	/	^118^
	SQSTM1: Poly-ubiquitination (K63-type)	Accumulation of aberrant enlarged SQSTM1	Promoted autophagy	^119^

## Substrates of NEDD4-1: the roles of oncogenes

### PTEN

PTEN is primarily identified as a phosphatase for lipids, mainly phosphatidylinositol (PtdIns)-3,4,5-trisphosphate (PIP3), which antagonizes the activation of the proto-oncogenic PI3K-AKT-mTOR signaling pathway^42,43^. Because it is a haploinsufficient tumor suppressor^44,45^, subtle changes in PTEN affect tissue homeostasis and tumorigenesis; thus, the regulation of PTEN may emerge as a new target for the treatment of tumors. Disease progression caused by PTEN could be due to PTEN gene mutation, repression of PTEN gene expression, aberrant PTEN subcellular localization, or the PTM of PTEN, which interferes with PTEN activity.

PTEN is known to be ubiquitinated by WWP2, XIAP, and CHIP^46–48^. Poly-ubiquitinated PTEN is ubiquitinated and degraded intracellularly, and mono-ubiquitinated PTEN is regulated in the nucleus^49^. NEDD4-1 was the first identified PTEN E3 ligase. Wang et al. found that NEDD4-1 negatively regulates PTEN, which promotes the poly-ubiquitination and degradation of PTEN in the cell. NEDD4-1 binds to PTEN through its C2 domain or HECT domain, whereas C-terminal truncated PTEN binds with higher affinity than full-length PTEN. Increasing levels of NEDD4-1 significantly reduce PTEN expression and potentiate cell proliferation and prostate tumorigenesis (Fig. 3a), suggesting the oncogenic role of NEDD4-1 in regulating PTEN functions^50,51^. A tissue microarray containing 103 non-small-cell lung carcinoma (NSCLC) resections revealed that NEDD4-1 is negatively correlated with PTEN protein, and the proliferation of NSCLC cells in vitro and in vivo was significantly inhibited due to the suppression of NEDD4-1 expression^52^. However, subsequent studies have shown no difference in the stability and localization of PTEN in two different strains of NEDD4-1-deficient mice, and PTEN expression is not related to NEDD4-1 expression in breast cancer^53,54^. Taken together, these findings favor the notion that NEDD4-1 promotes or inhibits tumorigenesis in different contexts.

Additional findings on the NEDD4-1-mediated ubiquitination of PTEN have implicated the importance of immunology, neurodegeneration and several Ub ligase adaptors and activator proteins. For example, Guo et al. found that NEDD4-1-mediated K63-linked poly-ubiquitination of PTEN at the lysine 13 site induces PTEN inactivation in Cbl-b(−/−) T cells^55^. As a neurodevelopment-related E3, NEDD4-1 is prominently expressed in axon growth cones, where it promotes terminal branching by downregulating PTEN^56^. However, Hsia et al. reported that NEDD4-1 is required for axonal growth but not involved in the ubiquitination of PTEN. Kwak found that zinc treatment decreases PTEN and increases NEDD4-1 expression in parallel in neurons and that NEDD4-1 is the major E3 ligase responsible for PTEN ubiquitination^57^. Furthermore, Ndfip proteins have been proposed to act as potent adaptors for NEDD4 family Ub ligases on endosomal membranes through binding to the WW domains. Ndfip proteins also function as adaptor proteins by promoting the association between the ligase and its substrate, including NEDD4-1 and PTEN^58^. The adaptor protein Numb was recently found to be a binding partner of NEDD4-1 that controls NEDD4-1 activity to modulate PTEN ubiquitination^59^.

### MDM2

The human homolog of murine double minute 2 (HDM2) and the highly homologous protein MDM2, are used interchangeably^60^. HDM2 and MDM2 negatively regulate the tumor suppressor gene P53, and overexpression of MDM2 leads to the inactivation of P53 function. MDM2 regulates P53 by two mechanisms; it acts as a RING E3 Ub ligase that targets p53 for ubiquitination and proteasomal degradation and inhibits the transcriptional activation domain. MDM also acts as a P53-independent oncogene and promotes neoangiogenesis^61^. A recent report indicated that MDM2 stability can be regulated by auto-ubiquitination of its own RING domain; in addition, other E3 ligases, such as Skp-cullin-F-box E3 complex (SCF^β-TRCP^) and PCAF (which has intrinsic E3 activity), can promote MDM2 degradation^62–64^. Wang et al. found that recombinant NEDD4-1 promotes K63-type poly-ubiquitination of MDM2 in a concentration- and time-dependent manner via the RING domain of MDM2. NEDD4-1 knockdown shortens the half-life of MDM2 and increases the level and activity of P53, resulting in enhanced P53 responses to DNA damage and inhibition of proliferation (Fig. 3a)^33^.

### Cyclic nucleotide ras GEF (CNrasGEF)

CNrasGEF, a guanine-nucleotide exchange factor in the small GTPase Ras superfamily, is expressed mainly in the brain and localized at the plasma membrane^65^. A C-terminal fragment of CNrasGEF was isolated via an expression screen of a library derived from a 16-day-old mouse embryo with the second WW domain of NEDD4-1 used as a probe. CNrasGEF binds with NEDD4-1 via the PY motifs of CNrasGEF and the WW domains of NEDD4-1. NEDD4-1 promotes the ubiquitination of CNrasGEF and targets CNrasGEF for proteasomal degradation^66^. Previous studies suggest that the role of CNrasGEF in tumor biology is complicated. For instance, CNrasGEF was verified to inhibit melanogenesis and cell survival in melanoma cells^67^. Zhang found that NEDD4-1 promoted but CNrasGEF inhibited the migration and invasion of glioma cells. NEDD4-1 ubiquitinated CNrasGEF and post-translationally modified CNrasGEF in glioma tissues. Furthermore, CNrasGEF downregulation enhanced the effect of NEDD4-1 overexpression on cell migration and invasion (Fig. 3a). CNrasGEF was demonstrated to mediate the role of NEDD4-1 as a substrate^68^.

### FGFR1

Fibroblast growth factor receptor 1 (FGFR1) plays critical roles in regulating cell proliferation, differentiation and animal development^69^. Ligand (FGF) binding to FGFR1 (along with heparin/HSPG binding) induces receptor dimerization, activating FGFR1 kinase activity and further enhancing intracellular signaling, such as PI3K/Akt and Ras/Erk signaling^70^. In contrast, phosphorylation of FGFR1 removes the protein from the plasma membrane and causes its subsequent lysosomal degradation^71^. Persaud found that NEDD4-1 binds directly to and ubiquitinates activated FGFR1 via the WW3 domain of NEDD4-1 and a novel noncanonical sequence (non-PY motif) on FGFR1. This binding and ubiquitination were completely abolished in the FGFR1-Δ6 mutant; this mutation seemed to stabilize active FGFR1 and enhance downstream signaling (Akt, Erk1/2). As a membrane protein, NEDD4-1 promotes the endocytosis of active FGFR1^72^. Furthermore, the same author, Dr. Persaud, demonstrated that the activation of FGFR1 (by factors such as epidermal growth factor (EGF)) led to c-Src kinase-dependent tyrosine phosphorylation of NEDD4-1, enhancing the Ub ligase activity of NEDD4-1. These findings may suggest a feedback mechanism involving receptor crosstalk (Fig. 3a)^73^. Additionally, Attali et al. discovered the conservation of auto-ubiquitination-dependent NEDD4-1 oligomerization and demonstrated the effect of NEDD4-1 on FGFR1^74^. Trimerization inactivated the ligase. The replacement of ubiquitinated lysine by arginine impairs this mechanism of inactivation and leads to unrestricted ubiquitination of FGFR1 in cells.

## Substrates of NEDD4-1: the roles of tumor suppressor genes

### N-Myc

N-Myc is a member of the Myc transcription factor family whose enhanced and dysregulated expression drives the development of a variety of tumors, including tumors of the nervous, blood systems, and neuroendocrine systems. N-Myc, a short-lived protein, is known to be ubiquitinated by FBXW7, HUWE1, and TRUSS^75–77^. In an Affymetrix gene array study in human neuroblastoma cells 30 h after transfection with scrambled control siRNA or SIRT2 siRNA-1, the gene most notably reactivated by SIRT2 siRNA-1 was NEDD4-1. SIRT2 transcriptionally inhibits NEDD4-1 by directly binding to the promoter of NEDD4-1 and deacetylating histone H4K16. Importantly, NEDD4-1 directly binds to Myc in the nucleus and negatively regulates N-Myc protein stability by increasing its poly-ubiquitination. Small molecule SIRT2 inhibitors were shown to activate NEDD4-1, reduce N-Myc protein expression, and inhibit neuroblastoma cancer cell proliferation (Fig. 3b)^78^.

### Her3

HER3, a member of the epidermal growth factor receptor (EGFR) family^79^, has been reported to increase cell migration, proliferation and poor prognosis in various cancers^80^. EGFR family receptors have been shown to be ubiquitinated and trafficked to proteasomes or lysosomes^81^. NEDD4-1 is an E3 ligase that acts on HER3, and the WW domains of NEDD4-1 interact with the C-terminal tail of HER3. Overexpression of NEDD4-1 decreases HER3 levels and increases HER3 ubiquitination. Conversely, downregulation of NEDD4-1 increases HER3 levels, resulting in enhanced HER3 signaling^82^. Low NEDD4-1 levels enhance HER3-mediated tumor growth in vivo and cell proliferation and migration in vitro. More importantly, cancer cells with NEDD4-1 inhibition exhibit increased sensitivity to anti- HER3 antibody treatments (Fig. 3b)^82^.

### SAG

Sensitive to apoptosis gene (SAG), an antiapoptotic protein, protects cells from apoptosis induced by various stimuli^83,84^. SAG is also an E3 Ub ligase that belongs to the RING component of SCF, which ubiquitinates and degrades many protein substrates. A large-scale proteomic study identified SAG as a putative NEDD4-1 binding partner^40^, which was verified by Zhou et al., who reported that NEDD4-1 directly binds with the C-terminal RING domain of SAG via its HECT domain and ubiquitylates SAG for proteasomal degradation. Functionally, ectopic NEDD4-1 expression sensitizes cancer cells to etoposide-induced apoptosis by decreasing SAG levels (Fig. 3b)^28^.

### Akt

Akt is a vital PTEN/PI3K/Akt signaling hub that regulates cell metabolism, cell cycle progression, proliferation, and differentiation by regulating more than 100 downstream target substrates^85^. Recent studies have reported that steady-state levels of Akt can also be ubiquitinated. The RING finger family E3 ligase TRAF6 ubiquitinates Akt and increases Akt membrane recruitment and phosphorylation, which are dependent on growth factor stimulation^86^. Furthermore, NEDD4-1 is an E3 ligase targeting Akt for phosphorylation and nuclear trafficking in the IGF-1 response. NEDD4-1-mediated Akt K63-linked ubiquitination requires the HECT domain of NEDD4-1 and the PH domain of Akt, but Akt ubiquitination is independent of its phosphorylation status. NEDD4-1-mediated ubiquitination regulates the levels of pAKT but not total Akt^87,88^. We found that NEDD4-1 enhances the sensitivity of multiple myeloma to bortezomib via attenuating the PTEN/PI3K/Akt signaling pathway in vivo and in vitro. Furthermore, NEDD4-1 was found to bind directly to Akt and ubiquitinate pAkt-Ser473 for proteasomal degradation (Fig. 3b)^89^. In contrast, Huang et al. found that depletion of NEDD4-1 reduces pAKT levels, increases PTEN levels and suppresses the growth and migration of hepatocellular carcinoma (HCC) cells^90^.

### Ras

Ras mutations are found in ~30% of all human cancers and in up to 90% of pancreatic cancers and 50% of colorectal and thyroid cancers, establishing RAS genes as commonly mutated proto-oncogenes in tumors^91,92^. However, the mechanisms regulating Ras stability remain generally unknown. There is a negative feedback loop between NEDD4-1 and Ras; Ras signaling stimulates the transcription of NEDD4-1, which in turn acts as an E3 Ub ligase that mediates Ras levels. Persistently activated Ras proteins block NEDD4-1-mediated Ras degradation and ubiquitination and enhance PTEN degradation through NEDD4-1, therefore enabling Ras proteins to escape the control of NEDD4-1, which might be essential for Ras-driven tumorigenesis (Fig. 3b)^93^.

## Substrates of NEDD4-1: others

### Notch

As a Ub ligase, NEDD4-1 plays a key role in the endocytosis of membrane proteins^94^. The Notch gene encodes a highly conserved cell surface receptor that allows cells to communicate with one another and regulate cell development and homeostasis. According to the tissue and cellular context, the Notch signaling pathway can inhibit or promote tumors. Invertebrate models such as nematodes and *Drosophila* are often used to study the function and mechanism of Notch because of Notch is highly evolutionarily conserved. Using the *Drosophila* system, NEDD4-1 was found to ubiquitinate Notch through the PPSY motif of Notch to reduce its stability. Loss of NEDD4-1 function inhibits the Notch and Deltex mutant phenotypes, and NEDD4-1 overactivation attenuates Notch activity. In tissue culture cells, enzyme-inactivated NEDD4-1 blocks the homeostasis of Notch internalization and activates Notch signaling independent of ligand binding. Transcriptional activation of the NEDD4-1 locus results in a nicked wing phenotype in the adult wing called the Notch-like wing defect (Fig. 3c)^95^. In addition, Baron found that coexpressing Notch with DNEDD4^C-A^ resulted in a synergistic increase in Notch signaling^96^. Kandarian et al. first elucidated the Ub ligase properties of NEDD4-1 in muscle. The expression of NEDD4-1 mRNA in the soleus muscle of rats increased after 1–14 days of hindlimb unloading, and NEDD4-1 mediated the inactivation of Notch1. Enzyme-inactivated NEDD4-1 reversed the reduction in Notch caused by unloading^97^. In 2012, another study demonstrated increased Notch-1 in denervated muscle and that there was no change in the magnitude of the increase in cleaved Notch-1 between NEDD4-1 SMS-knockout (KO) (NEDD4-1 skeletal muscle-specific KO mouse) and littermate control mice. There is no evidence that NEDD4-1 is involved in Notch-1-mediated, denervation-induced skeletal muscle atrophy^98^.

### RNAPII

One interesting substrate of protein ubiquitination is RNA polymerase II (RNAPII), which transcribes all protein-encoding genes into mRNA in three stages: initiation, elongation, and termination^99^. Ubiquitination and degradation of the largest subunit of RNAPII (Rpb1) are known to occur in UV-irradiated human cells^100^. Other experiments have shown that RNAPII arrested during transcription elongation due to other transcription obstacles is also susceptible to ubiquitination and degradation^101^. In *S. cerevisiae*, Rsp5 is the E3 responsible for RNAPII ubiquitination. NEDD4-1 has the highest sequence homology, exceeding that of five other Rsp5 homologs, to Rsp5 (52% identity, 69% similarity) in humans^19^. NEDD4-1 is an E3 that is indeed required for Rpb1 ubiquitination and degradation in response to UV-induced DNA damage, and proteasome inhibitors prevented this degradation. Reduced levels of NEDD4-1 impaired RNAPII ubiquitination. However, the activity of the remaining NEDD4-1 was quickly restored, and RNAPII was reubiquitinated. Furthermore, NEDD4-1 coimmunoprecipitated with RNAPII from chromatin, and with further UV exposure, an increasing amount of NEDD4-1 was associated with RNAPII^102^. Interestingly, Harreman found that two distinct E3s cooperate via a two-step mechanism to poly-ubiquitinate RNAPII for its degradation. First, Rsp5 produces a mixture of mono-ubiquitinated and K63-linked poly-ubiquitinated RNAPII, but only the poly-ubiquitinated RNAPII is not degraded. Then, the Elongin-Cullin complex produces a Ub chain linked via K48, which can trigger proteolysis (Fig. 3c)^103^.

### N4BP

A yeast two-hybrid screen carried out by Murillas et al. identified four proteins expressed in the midgestation embryo that are able to interact with NEDD4-1, namely, NEDD4-1 binding partners 1–4 (N4BP1-4)^37^. N4BP1-3 binds NEDD4-1 in vitro, but only N4BP1 and N4BP2 are ubiquitinated by NEDD4-1. N4BP1 is the first nonviral protein recognized as a substrate of NEDD4-1 for mono-ubiquitination. The proline-rich domains of N4BP1 that interact with the WW domains of NEDD4-1 are dispensable for the interaction between N4BP1 and NEDD4-1. The disruption of three proline-rich regions of N4BP1 had no effect on the in vitro binding of N4BP1 to NEDD4-1. In addition, N4BP1 is mono-ubiquitinated by NEDD4-1 in vivo, while N4BP2 is poly-ubiquitinated in vivo, and the level of N4BP2 but not that of N4BP1 was increased by proteasome inhibition^37^. Later, the authors found that NEDD4-1 can also mediate the poly-ubiquitination and proteasomal degradation of N4BP1 in promyelocytic leukemia nuclear bodies (Fig. 3c)^104^. Furthermore, N4BP1 functions as a negative regulator of Itch, an E3 structurally related to NEDD4-1 that contains four WW domains, and endows it with substrate-binding activity^105^.

### α-Synuclein

The diffuse accumulation of α-synuclein in Lewy bodies is one of the main pathological features of Parkinson’s disease (PD)^106^. Tofaris et al. found that NEDD4-1 is expressed strongly in neurons containing Lewy bodies and that NEDD4-1 downregulation impaired the degradation of α-synuclein (Fig. 3c). The C-terminus of α-synuclein contains a proline-rich (PxY, PS) motif, **P**VD**P**DNEAYEM**P**SEEGYQDYE**P**EA, that is specifically recognized by members of the NEDD4 family. Seven different mutant Ubs that contained only one lysine residue were produced, and NEDD4-1 could mono-ubiquitinate α-synuclein with nearly all of these Ub mutants but promoted only K63-linked long poly-ubiquitination. Surprisingly, ubiquitination by NEDD4-1 targets α-synuclein to endosomes and lysosomes but not to the proteasome^107^. The above results indicate that an increase in NEDD4-1 is characteristic of a protective response that assists in reducing the accumulation of potentially toxic proteins and helps protect against the pathogenesis of Parkinson’s disease.

### ABCB1

Multidrug resistance (MDR) to pharmaceutical active agents is a universal clinical problem in patients with tumors. The ATP-binding cassette transporter ABCB1 is an MDR-associated transporter. The inhibition of ABC transporters in cancer patients has been extensively investigated in clinical trials^108^. In patients with Alzheimer’s disease, the level of ABCB1 and accumulation of the neurotoxic peptide β-amyloid in the brain are correlated. PTM with Ub was reported to involve internalization of the transporter from the abluminal membrane. Human ABCB1 was demonstrated to be a substrate of E3 NEDD4-1 in vitro. Mass spectrometry identified eight lysine residues of ABCB1—K271, K272, K575, K685, K877, K885, K887, and K1062—that were ubiquitinated by NEDD4-1. Transient expression of NEDD4-1 in HEK293 cells stably expressing ABCB1 was shown to decrease the surface level of the transporter (Fig. 3c)^109^.

### Autophagy-related proteins: Beclin 1, LC3, and p62

Autophagy is an important cellular process that releases cells from stress conditions, such as endoplasmic reticulum (ER) stress, nutritional starvation, and mitochondrial damage. A variety of E3s have been implicated in the regulation of autophagy via PTM^110^. NEDD4-1 was demonstrated to promote autophagy in human prostate carcinoma. mTOR signaling was suggested to be involved in NEDD4-1-mediated autophagy due to decreased NEDD4-1 expression and notably increased activated mTOR (p-mTOR) levels^111^.

Beclin 1 is thought to form the core of the PI3K-III complex. Decreased Beclin 1 levels are associated with the occurrence of cancer^112^, but increased Beclin 1 levels are correlated with the prolonged survival of tumor cells, most likely by enhancing autophagy and preventing apoptosis^113^. Beclin 1 contains a sequence resembling a PY motif (LPxY), and the WW domains of NEDD4-1 interact specifically with this motif in Beclin 1. NEDD4-1 facilitates the K11- and K63-linked poly-ubiquitination of Beclin 1 and controls the stability and proteasomal degradation of Beclin 1 via K11-linked poly-ubiquitination, which then inhibits autophagy (Fig. 3c). Notably, Beclin 1 was the first tumor suppressor demonstrated to be controlled by K11-linked poly-ubiquitination^114^.

Microtubule-associated protein 1 light chain 3 (MAP1LC3), also known as LC3, is essential in autophagy and involved in the biogenesis and transport of autophagosomes^115^. LC3 conjugated to phosphatidylethanolamine (PE) was shown to bind to LC3-II, which is thought to be a marker of autophagosomes, and the formation of LC3-II can be used as an index of autophagic activity^116^. SQSTM1 is a common autophagic cargo receptor involved in various types of selective autophagy^117^. Sun and others showed that NEDD4-1 binds to LC3 through a conserved LIR domain and is involved in autophagosome biogenesis. Downregulation of NEDD4-1 weakened starvation- or rapamycin-induced autophagy and autophagosome formation and induced the aggregation of LC3 puncta colocalized with ER membrane markers. Depletion of NEDD4-1 decreased the level of LC3 and increased the level of SQSTM1. Surprisingly, LC3 is not a substrate of NEDD4-1 ubiquitination. When LC3B was coexpressed with NEDD4-1 in HEK293 cells and isolated by either immunoprecipitation or GST-UBA pulldown, no ubiquitinated LC3B was detected^118^. However, SQSTM1 is a K63-linked ubiquitination substrate of NEDD4-1 in cells, while LC3 binding might activate NEDD4-1 ligase activity. NEDD4-1 interacts with and ubiquitinates the PB1 domain of SQSTM1 via its HECT domain (Fig. 3c)^119^.

### Clinical relevance and therapeutic strategies

Aberrant NEDD4-1 expression has been frequently observed in various tumors. NEDD4-1 acts as an oncogene as well as a tumor suppressor in cancers^49,52,78,93,120–128^, and NEDD4-1 activators or inhibitors are urgently needed. Stefan et al. discovered and structurally identified the first covalent inhibitor of NEDD4-1, which switches the mechanism of NEDD4-1 from processive to distributive, following which NEDD4-1 synthesizes the attachment of poly-ubiquitin chains to the substrate in the presence of the deubiquitinating enzyme USP8^129^. The chemopreventive agent indole-3-carbinol (I3C), a natural indole carbinol compound derived from cruciferous vegetables such as broccoli and Brussels sprouts, directly binds purified NEDD4-1 protein and interrupts the ubiquitination and proteasomal degradation of PTEN by NEDD4-1 without altering its transcript levels. The above mechanism is involved in the antiproliferative response of I3C in human melanoma cells. NEDD4-1 was identified as a new biologically significant I3C target protein^130^. Furthermore, 1-benzyl-I3C has a lower IC50 and is a significantly more potent enzymatic inhibitor of NEDD4-1 than I3C. Protein thermal shift assays combined with in silico binding simulations showed that each of the indole carbinol compounds interact with the purified catalytic HECT domain of NEDD4-1^131^. Moreover, Jing found that exosomal hsa-miR199a-3p promotes tumor proliferation and migration via inhibiting NEDD4-1 expression by binding to NEDD4-1 mRNA in neuroblastoma^132^. In addition, a bicyclic peptide isolated by phage display, termed heclin (a HECT ligase inhibitor), can target the E2-binding sites in the HECT domains of NEDD4-1, Smurf2, Huwe1, and WWP1 and inhibit ligase activity. Heclin causes a conformational change that results in oxidation of the active site Cys but does not block E2 binding. Furthermore, exposure to heclin for 24 h led to the death of growing HEK293 cells, which may be due to the effect of HECT ligase inhibition on many important cellular processes^133^. However, exactly which HECT ligases are essential remains unknown.

PTEN is a well-known substrate of NEDD4-1. p34 was shown to interact with the WW1 domain of NEDD4-1 via the SERTA domain, which contains a proline-rich region (PRR motif); this interaction promotes PTEN poly-ubiquitination, leading to PTEN protein degradation. Knockdown of p34 results in PTEN mono-ubiquitination, suggesting that p34 controls a switch between NEDD4-1-mediated mono- and poly-ubiquitination of PTEN. NEDD4-1 was previously demonstrated to be auto-ubiquitinated^50^, and p34 expression significantly potentiates tumorigenesis via decreasing NEDD4-1 auto-ubiquitination and stabilizing NEDD4-1^122,126,134^.

Biochemical, structural, and cellular analyses of NEDD4 family members showed that the WW domain and a following linker segment induce auto-inhibition of E3s, which can be relieved by linker phosphorylation^29–31^. Compared to full-length NEDD4-1, a NEDD4-1 mutant without the stretch of amino acids C-terminal to the WW1 domain (aa 225–244) showed enhanced auto-ubiquitination activity, indicating that the C2 domain and WW domain may synergize to auto-inhibit NEDD4-1 catalytic activity^29,31^. Removal of the C2 domain from NEDD4-1/4 L, Smurf1, and Smurf2 dramatically increased their enzymatic activity^135^. The above structural data also provide useful information for the design of new activators or inhibitors of NEDD4-1.

### Challenges, future prospects, and conclusion

Much progress has been made over the past few decades in identifying the multiple substrates of NEDD4-1 and elucidating the molecular mechanisms by which NEDD4-1 catalyzes substrate ubiquitination. The functional validation of specific ubiquitination sites and putative substrates in cellular and in vivo contexts still presents a major bottleneck and requires further study, although many suitable high-throughput biochemical methods, such as protein microarray, mass spectrometry, proteomics, and in silico computational modeling, are available^136^. This review discusses the role of the multifunctional NEDD4-1 protein in human cancer biology and the mode by which NEDD4-1 regulates each substrate. Most of the substrates of NEDD4-1 discovered thus far are poly-ubiquitinated or mono-ubiquitinated to various degrees (Table 1).

In fact, NEDD4-1 exhibits a dual role and acts as either an oncogene or tumor suppressor, which depends heavily on the context. NEDD4-1 is universally expressed in a diverse array of tissues; however, little is known about the regulation of NEDD4-1 expression in different tissues under physiological and pathological conditions. Furthermore, the nature of protein ubiquitination is dynamic. E3s and deubiquitinating enzymes (DUBs) tightly control ubiquitination in response to altered cellular environments. Finally, NEDD4-1 targets multiple substrates and in turn mediates many functions. This regulatory network between E3s and their substrates makes cellular analyses difficult and different.

The E3 ligase NEDD4-1 is a founding member of the NEDD4 family and is involved in cell proliferation, cell migration, cell differentiation, and tumorigenesis. Based on the many substrates and dual roles of NEDD4-1, strategies for blocking the interactions between NEDD4-1 and particular substrates with the fewest side effects may be more suitable for therapeutic treatment than strategies targeting NEDD4-1 activity directly, for example, attenuating the stabilization of MDM2 by NEDD4-1 (as shown in Fig. 3a) and enhancing the degradation of N-Myc by NEDD4-1 (as shown in Fig. 3b) to inhibit cancer cell proliferation. The advanced understanding of the different types of Ub chains used by NEDD4-1 to regulate its specific downstream targets may contribute to comprehending the mechanism underlying metastasis and tumorigenesis. In general, although the study of NEDD4-1 is still in its infancy, knowledge of NEDD4-1 has the potential to be applied to translational studies to greatly impact human health.

## Data Availability

All datasets and materials generated and/or analyzed during the current study are available.
